# miRNAs as biomarkers of autism spectrum disorder: a systematic review and meta-analysis

**DOI:** 10.1007/s00787-023-02138-3

**Published:** 2023-02-03

**Authors:** Nathalia Garrido-Torres, Karem Guzmán-Torres, Susana García-Cerro, Gladys Pinilla Bermúdez, Claudia Cruz-Baquero, Hansel Ochoa, Diego García-González, Manuel Canal-Rivero, Benedicto Crespo-Facorro, Miguel Ruiz-Veguilla

**Affiliations:** 1 Hospital Universitario Virgen Del Rocio, IBIS-CSIC, Department of Psychiatry, University of Sevilla, Avda Manuel Siurot S/N, 41013 Seville, Spain; 2 Spanish Network for Research in Mental Health (CIBERSAM), Seville, Spain; 3 Colegio Mayor de Cundinamarca, Bogotá, Colombia; 4 Epidemiology Research Group (EpiAndes), Los Andes University, Bogotá, Colombia

**Keywords:** Autism spectrum disorder, microRNA, Biomarker, Saliva

## Abstract

Autism spectrum disorder (ASD) is a neurodevelopmental disorder with complex clinical manifestations that arise between 18 and 36 months of age. Social interaction deficiencies, a restricted range of interests, and repetitive stereotyped behaviors are characteristics which are sometimes difficult to detect early. Several studies show that microRNAs (miRs/miRNAs) are strongly implicated in the development of the disorder and affect the expression of genes related to different neurological pathways involved in ASD. The present systematic review and meta-analysis addresses the current status of miRNA studies in different body fluids and the most frequently dysregulated miRNAs in patients with ASD. We used a combined approach to summarize miRNA fold changes in different studies using the mean values. In addition, we summarized *p* values for differential miRNA expression using the Fisher method. Our literature search yielded a total of 133 relevant articles, 27 of which were selected for qualitative analysis based on the inclusion and exclusion criteria, and 16 studies evaluating miRNAs whose data were completely reported were ultimately included in the meta-analysis. The most frequently dysregulated miRNAs across the analyzed studies were miR-451a, miR-144-3p, miR-23b, miR-106b, miR150-5p, miR320a, miR92a-2-5p, and miR486-3p. Among the most dysregulated miRNAs in individuals with ASD, miR-451a is the most relevant to clinical practice and is associated with impaired social interaction. Other miRNAs, including miR19a-3p, miR-494, miR-142-3p, miR-3687, and miR-27a-3p, are differentially expressed in various tissues and body fluids of patients with ASD. Therefore, all these miRNAs can be considered candidates for ASD biomarkers. Saliva may be the optimal biological fluid for miRNA measurements, because it is easy to collect from children compared to other biological fluids.

**Supplementary Information:**

The online version contains supplementary material available at 10.1007/s00787-023-02138-3.

## Introduction

Autism spectrum disorder (ASD) is a neurodevelopmental disorder with a prevalence of approximately 1.5% in developed countries [1]. The aetiology of ASD has not been fully elucidated, since the disorder exhibits wide genetic variability that triggers both behavioral and phenotypic alterations at the level of brain structures. The clinical manifestations are complex and emerge between 18 and 36 months of age. Social interaction deficiencies, a restricted range of interests, and repetitive stereotyped behaviors are the main characteristics of ASD, which are sometimes difficult to detect early [2]. Currently, the diagnosis is based on interviews with parents or caregivers using tools such as the Modified Checklist for Autism in Young Children, Revised (M-CHAT-R) [3], the Autism Diagnostic Observation Schedule (ADOS) [4], and the Autism Diagnostic Interview-Revised (ADI-R) [5]. Although the diagnostic reliability of these interviews is high, these tools require an evaluator with experience and specific training.

Implementation of an intervention before the age of two can improve the prognosis. Early intervention can also lead to better neuronal maturation [6]. Therefore, a biomarker for early detection would be a good diagnostic supplement [7, 8]. Several biomarkers have been proposed for ASD detection, including functional connectivity observed on magnetic resonance imaging and calculated using machine learning algorithms [9], the genetic load [10], the increased CSF volume [11], transcriptomic signatures in blood, and levels of altered cytokines; however, more studies are required to corroborate whether these items can serve as ideal biomarkers [12].

The non-coding RNAs (ncRNAs) are recently emerging as novel promising biomarkers in medicine with great prognostic and predictive potential [13]. MiRNAs, the most well-studied ncRNA, are short non-coding RNAs of approximately 18–24 nucleotides that are responsible for regulating gene expression through epigenetic mechanisms [14] in approximately 60% of human genes [15]. As will be focused on this review, for ASD diagnoses, microRNAs (miRs/miRNAs) are types of ncRNAs which have become an important research focus last years [15–17].

MiRNAs, the most well-studied ncRNA, are short non-coding RNAs of approximately 18–24 nucleotides that are responsible for regulating gene expression through epigenetic mechanisms [14] in approximately 60% of human genes [15]. In addition, miRNAs are heavily involved in neuronal plasticity and neuronal development [18], and their deregulation generates diverse neurological alterations, such as ASD. To be an accessible biomarker, miRNA should be able to be isolated using non-invasive protocols, easy to quantify, specific to the disorder, able to be translated from systems to human models [19] and reliable as an early indicator of disease onset [20]. Various investigations of miRNAs as biomarkers for ASD have focused on biogenesis and measurement in different biofluids or tissues for detection [21], such as lymphoblastoid cells [22, 23], postmortem cerebral cortex tissues [2], serum or blood plasma [24], olfactory mucosa cells [18], and saliva [16].

Based on this knowledge, we conducted a systematic review to (1) identify which miRNAs can be used as biomarkers to support current diagnostic methods, (2) determine which body fluid may be ideal for miRNA measurement in children, and (3) clarify relationships between miRNAs and the genetic burden of ASD.

## Methodology

The systematic review was conducted in accordance with the Preferred Reporting Items for Systematic Reviews and Meta-Analyses (PRISMA) statement [25]. It also followed a protocol registered in PROSPERO CRD42021225956.

### Search strategy

We searched the PubMed, Scopus, and Web of Science databases from 2010 until March 2021 utilizing Medical Subject Headings (MeSH) related to (autism spectrum disorder) AND (microRNA). We also cross-referenced relevant studies and previous reviews and contacted study authors and experts for data clarification.

### Eligibility

To achieve a concise and precise review of the role of miRNAs in ASD, some criteria were selected to systematize the search in the different databases and thus include only articles related to the subject. Inclusion criteria: (i) Studies on ASD patients; (ii) Studies in which ASD was diagnosed according to either the ADOS, ADI-R or any validated instrument; (iii) Studies measuring miRNAs in any peripheral tissue from patients with ASD; (iv) Studies measuring miRNAs in CNS from patients with ASD (iv) Studies with a control group. Exclusion criteria: (i) ASD studies focusing on biomarkers other than miRNAs; (ii) Clinical cases reports; (iii) Studies conducted 11 or more years ago; (iv) Studies conducted in languages other than English; (v) Review articles; and (vi) Studies conducted on animals.

### Study selection

Three independent reviewers (KGT, NGT, and GPB) screened titles and abstracts to identify studies meeting the inclusion criteria outlined above using Rayyan [26] software. The same reviewers then reviewed the full texts of eligible articles, and the final list of included articles was established through consensus. The Kappa index was 0.931. Disagreements regarding the eligibility of studies were resolved through discussions with two additional reviewers (SGC and CCB).

### Meta-analytic approach

We implemented a meta-analytic approach to combine the differential gene expression results of the included studies. After study selection, the reviewers used a predefined spreadsheet to extract all relevant data from the included studies. The extracted data included general publication information and detailed information about the miRNAs analyzed in each study and their association with severity and clinical manifestations (impaired social behavior, repetitive behaviors, intelligence, memory and learning, and language impairment). We extracted fold change (fc) data or log2-fc values and their *p* values and confidence intervals when reported by the authors. We identified miRNAs that were assessed and reported in two or more studies to combine their log2-fc values. We used the R package MetaVolcanoR, which implements three strategies to combine and summarize differential gene expression data from different studies: a random-effects model approach, a combined *p* value approach, and a vote-counting approach [27].

### Quality assessment

The QUADAS-2 tool was developed to assess the quality of studies on diagnostic tests included in systematic reviews [28]. QUADAS-2 analyzes four domains: (1) patient selection, (2) index tests, (3) reference tests, and (4) flow and times. Each domain is evaluated in terms of its risk of bias, and the first three domains are also evaluated for their applicability. For more details, please see the supplementary documents.

## Results

**Fig. 1:**
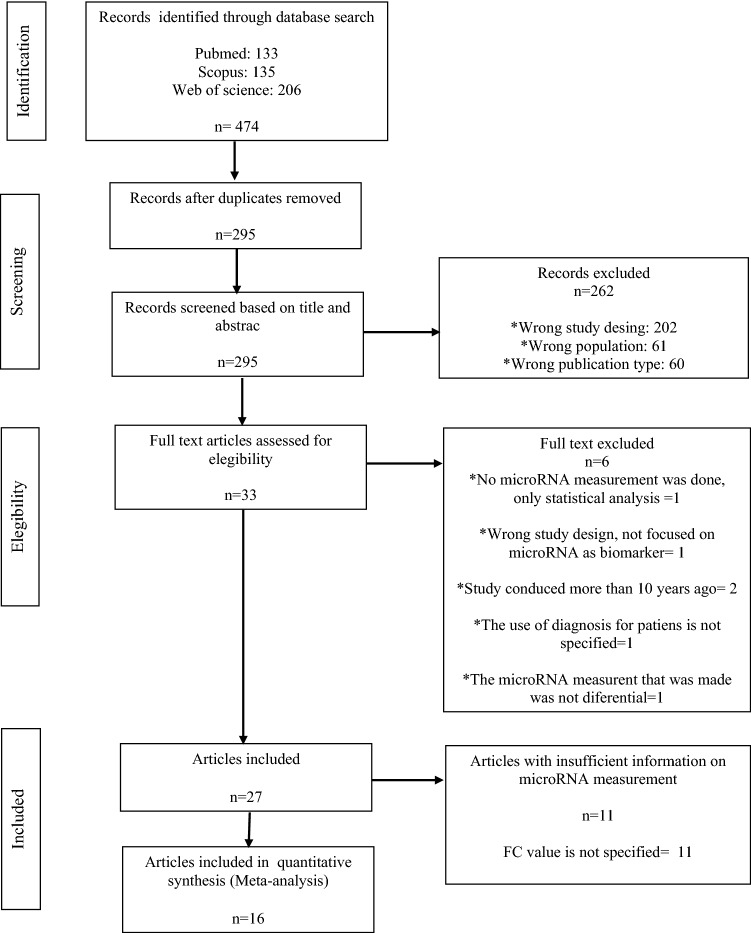
PRISMA diagram

The literature search yielded a total of 133 articles. Then, 27 articles were selected for qualitative analysis based on the inclusion and exclusion criteria. Out of the 27, 16 studies were ultimately included in the meta-analysis and 218 miRNAs were identified (Fig. 1).

In all studies (Tables 1, 2), miRNA measurements were performed in ASD patients and compared with miRNA measurements in a control group. The ages of the patients and controls ranged between 2 and 81 years. miRNA was measured in lymphoblastoid cell lines in three studies [14, 22, 29], five studies measured miRNA in postmortem cerebral cortex tissues [2, 15, 30–32], one study used cells from the olfactory mucosa [18], thirteen studies used serum [7, 19, 24, 33–42], and five studies used saliva [8, 16, 43–45]. A recent narrative review [46] found that the miR-151a, miR-146a, and miR-27a-30 are dysregulated in people with ASD and are replicated in more than one tissue.

**Table 1:** Findings of the 26 studies included in the systematic review

Sample type	Number of patients M:F age (years)		Study type	Other patient characteristics	RNA extraction	miRNA profile and validation	Prediction of deregulated genes in ASD and microRNA target genes	References
	ASD (n)	CS (n)						
Cell lines	14 pts 14 M: 0 F 4–14 years old	14 pts (3 sets of brothers were monozygotic twins) 14 M: 0 F 2–12 years old	Experimental with a control group	Ethnicity of the patients: Hispanic or Latino Origin of the sample: Autism Genetics Resource Socioeconomic level: – Diagnostic criteria: ADI-R and ADOS	mirVana kit	Microarrays and qRT–PCR	miRBase, software prediction software Ingenuity Pathway Analysis (IPA) version 6.0 y Pathway Studio version 5 (Ariadne Genomics, Rockville, MD, USA)	Sarachana et al. [14]
	20 pts 3 M: 7 F Age: –	22 pts (brothers) 19 M: 3 F Age: –	Experimental with a control group	Ethnicity of the patients: European descent Origin of the sample: – Socioeconomic level: – Diagnostic criteria: ADOS	mirVana kit	Microarrays and qRT–PCR	Method of generalized estimation equations, to determine differentially expressed genes and network prediction software Ingenuity Pathway Analysis (IPA)	Seno et al. [22]
	10 pts 10 M:0 F 3–13 years old	17 pts 10 pts brothers 7 pts not brothers 17 M:0 F 4–13 years old	Experimental with control group	Ethnicity of the patients: EEUU Origin of the sample: Autism Genetic Resource Exchange and Coriell Cell Repository (Camden, NJ, USA) Socioeconomic level: – Diagnostic criteria: ADOS	miRNeasy Mini Kit (Qiagen, Valencia, CA, USA), and RNeasy MinElute Cleanup Kit was used for miRNA isolation (Qiagen, Valencia, CA, USA)	RNA-seq (Illumina Hiseq 4000) and qRT–PCR	TargetScan and Miranda 3.3a	Frye et al. [29]
Postmortem Brodmann area 10	12 pts 10 M:12 F 18–51 years old	12 pts 11 M:1F 16–44 years old	Experimental with a control group	Ethnicity of the patients: – Origin of the sample: Harvard Brain Bank except for two brain samples, which were obtained from the UK Brain Bank for Autism Socioeconomic level: – Diagnostic criteria: ADI-R PMI: 26 h	miRNeasy kit treated with ADNasa free of ARNasa	RNA-seq qRT–PCR	software DIANA-lab and microrna.org	Mor et al. [31]
Postmortem cerebral cortex tissue (PAC) with Brodman areas 41 and 42 and (STS) with Brodmann area 22	10 pts 5 M: 5 F 5–52 years old	8 pts 6 M:2 F 4–58 years old	Experimental with a control group	Ethnicity of the patients: – Sample Source: Harvard Brain Tissue Resource Center Socioeconomic level: – Diagnostic criteria: ADI-R PMI: 23.5 h for ASD PMI: 25 h for MC	Recover All	Microarrays ements	DIANA miRPath v2.0	Ander et al. [15]
Cerebral cortex tissue: frontal cortex FC, Brodmann area 9, temporal cortex and cerebellar vermis	131 pts 106 M: 25 F 8–81 years old	105 pts 88 M: 17 F 8–60 years old	Experimental with a control group	Ethnicity of the patients: White Northern European, Caucasian, Asian, English-White, African American/Black Origin of the samples: Harvard, NICHD, BBA, and the Brain Bank of Neurodegenerative Disorders at MRC Socioeconomic level: – Diagnostic criteria: ADI-R confirmation and ASD diagnosis supported by other evidence, such as clinical history PMI: 24.1 h	miRNeasy kit	RNA-seq qRT–PCR	Linear mixed effect model (LME) to determine gene deregulation and TargetScan to determine target genes for microRNA	Wu et al. [2]
Postmortem brain cut area 21 of Brodmann	5 pts 3 M: 2 F 4–9 years old	6 pts 4 M: 2 F 4–9 years old	Experimental with a control group	Ethnicity of the patients: African American and Caucasian Origin of the samples: NIH NeuroBioBank Socioeconomic level: – Diagnostic criteria: ADI-R, ADOS and DSM-IV PMI: (13–39) 14 h	miRVana™ miRNA isolation kit (AM1560, Thermo Fisher Scientific)	TaqMan assays on Fluidigm 98.98qPCR	miRTarBase miRDIP	Nguyen et al. [32]
Postmortem brain dorsolateral prefrontal cortex and Amygdala	15 pts 15 M: 0 F 6–12 years old	15 pts 15 M: 0 F 6–12 years old	Experimental with a control group	Ethnicity of the patients: – Origin of the samples: NIH NeuroBioBank at the University of Maryland, Baltimore MD Socioeconomic level: – Diagnostic criteria: – PMI: (10–24 h) for ASD PMI: (12–18 h) for MC	themirVana™miRNA isolation kit (Ambion, Life Technologies)	QRT-PCR	–	Almehmadi et al. [30]
Stem cells of the olfactory mucous membrane	10 pts 7 M: 3F 22–43 Years old	10 pts 7 M: 3F 18–44 Years old	Experimental with a control group	Ethnicity of the patients: French Origin of the samples: Hospitals, medical social support centers for ASD and private practices in France Socioeconomic level: – Diagnostic criteria: DSM-5/ICD-10	mirVana kit	qPCR high performance real time	mir-DIP and software of network prediction Ingenuity Pathway Analysis (IPA)	Nguyen et al. [18]
Serum	55 pts 48 M: 7 F 6–16 years old	55 pts 48 M: 7 F 6–16 years old	Experimental with a control group	Ethnicity of the patients: Japanese Origin of the samples: – Socioeconomic level: – Diagnostic criteria: DSM-4 and ADI-R	Qiagen miReasy serum/plasma kit	miRNA PCR and qRT–PCR	DIANA-mirPath v2.0	Vasu et al. [40]
	20 pts 17 M: 3 F 2.5–7 years old	20 pts - M: -F 2.5–7 years old	Experimental with a control group	Ethnicity of the patients: Chinese Origin of the samples: Xiangya Hospital China Socioeconomic level: – Diagnostic Criteria: DSM-4	TRIzol life	Microarrays and qRT–PCR	TargetScan, miRanda, CLIP-Seq and miRDB	Huang et al. [34]
	30 pts 24 M: 6 F 3–11 years old	30 pts 24 M: 6 F 3–11 years old	Experimental with a control group	Ethnicity of the patients: Bulgarian Origin of the samples: University Hospital of Plovdiv, Bulgaria Socioeconomic level: – Diagnostic criteria: ADI-R, CARS, GARS, and DSM-5	PAXgene blood miRNA kit	qRT–PCR	miRWalk Data base	Kichukova et al. [36]
	30 ASD 22 M: 8 F 25 ASD + ST 16 M: 9 F 24 ST 21 M: 3 F 3–13 years old	25 pts 25 M: 0 F 3–13 years old	Experimental with a control group	Ethnicity of the patients: – Origin of the samples: Section of Child and Adolescent Psychiatry, Department of Clinical and Experimental Medicine, University of Catania Socioeconomic level: Diverse Diagnostic criteria: ADOS, DMS-5, and ADI-R	Qiagen miReasy kit serum/plasma	TLDA and qRT–PCR	DIANA-TarBase v7.0 y	Cirnigliaro et al. [33]
	69 pts 52 M: 16 F 2.8–27 years old	27 pst 16 M: 11 F 3.6–27 years old	Experimental with a control group	Ethnicity of the patients: African American, Asian and Caucasian Origin of the samples: Paediatric Allergy/Immunology Clinic Socioeconomic level: – Diagnostic criteria: ADOS, ADI-R and Vineland Adaptive Behavior Scale	miRNAeasy kit	Ion Total RNA-Seq Kit V2, Ion One Touch 2 system (Life Technologies) and Ion 318 chips (Life Technologies)	mirDIP	Jyonouchi et al. [35]
	7 pts 7 M: 0 F 7.5 (mean age)	4 pts 4 M: 0 F 7.5 (mean age)	Experimental with a control group	Ethnicity of the patients: – Origin of the samples: Hospital de Clínicas de Porto Alegre Brazil Socioeconomic level: – Diagnostic criteria: DSM-5, CARS, and ADOS	Invitrogen, TRIzol	qRT–PCR	miRBase	Vaccaro et al. [39]
	20 pts 18 M: 2 F 3–9 years old	23 pts 20 M: 3 F 3–9 years old	Experimental with a control group	Ethnicity of the patients: Chinese Origin of the samples: Zhongnan Hospital of Wuhan University Socioeconomic level: – Diagnostic criteria: DSM-5	mirVana kit	Microarrays and qRT–PCR	–	Yu et al. [41]
	30 pts 24 M: 6 F 3–11 years old	30 pts 24 M: 6 F 3–11 years old	Experimental with a control group	Ethnicity of the patients: Bulgarian Origin of the samples: Plovdiv Medical University Socioeconomic level: – Diagnostic criteria: GARS, CARS, ADI-R, and DSM-5	PAXgene blood miRNA kit	qRT–PCR	miRWalk 2.0	Nt et al. [19]
	30 pts 18 M: 2F 7.3 (mean age)	30 pts 18 M: 2 F 8.4 (mean age)	Experimental with a control group	Ethnicity of the patients: Japanese Origin of the samples: Department of Psychiatry, Kyoto University Socioeconomic level: – Diagnostic criteria: WAIS, ADOS and SRS	Paxgene blood miRNA system	Microarrays and qRT–PCR	miRWalk 2.0	Nakata et al. [37]
	105 pts N.A 2.2–21.5 years old	34 pts 3.9–29–7 years old	Experimental with a control group	Ethnicity of the patients: African American, Asian, Caucasian and mixed. Origin of the samples: New Brunswick University Hospital, New Jersey, United States Socioeconomic level: – Diagnostic criteria: ADOS and ADI-R	Norgen Biotek Corp. kit	Deep sequencing with high performance	mirDIP y DAVID	Jyonouchi et al. [24]
	45 pts 31 M: 14 F 2–13 years old	21 pts 10 M: 11 F 3–16 years old	Experimental with a control group	Ethnicity of the patients: Turkish Origin of the samples: Erciyes University School of Medicine Hospital, Kayseri, Turkey Socioeconomic level: – Diagnostic criteria: DSM-IV and CARS	High Pure miRNA Isolation Kit (Cat. no: 5080576001; Roche, Mannheim, Germany)	qRT–PCR	DIANA-mirPath software	Ozkul et al. [38]
	37 pts 26 M: 11 F 3–15 years old	40 pts 27 M: 13 F 4–12 years old	Experimental with a control group	Ethnicity of the patients: Iranian Origin of the samples: Autism centers in two cities of Iran, including Tehran and Amol Socioeconomic level: – Diagnostic criteria: –	Precipitation method	qPCR	–	Atwan et al. [7]
	16 pts – M:– F < 14 years old	16 pts – M:–F < 14 years old	Experimental with a control group	Ethnicity of the patients: Egyptian Origin of the samples: Child Psychiatry Clinic, Paediatric Hospital, Ain Shams University Socioeconomic level: – Diagnostic criteria: DSM-V and CARS	miRNeasy kits (Qiagen, catalogue no. 217184, USA)	qRT–PCR	–	Zamil et al. [42]
Saliva	24 pts 19 M: 5 F 5–13 years old	21 Pts 16 M: 5 F 4–14 years old	Experimental with a control group	Ethnicity of the patients: – Origin of the samples: SUNY Center Socioeconomic level: – Diagnostic Criteria: DSM-5, ADOS, CARS and Vineland Adaptive Behavior Scale	Standard method with TRIzol and purification using the RNeasy mini column	RNA-seq	miRDB, DAVID, Simons Foundation Autism Database (AutDB)	Hicks et al. [44]
	238 ASD 201 M: 37 F 1.7–4 years old	218 pts 148 M: 10 F 1.7–4 years old	Experimental with a control group	Ethnicity of the patients: North American Origin of the samples: State University of New York, Upstate Medical University, Penn State Medical School and the University of California, Irvine Socioeconomic level: – Diagnostic Criteria: MCHAT-R/F, Vineland Adaptive Behavior Scale and DSM-5	Isolation of epithelial or exosomal RNA	RNA-seq	DIANA miRPath v3, Encyclopedia of Genes and Genomes of Kyoto and SFARI database	Hicks et al. [16]
	224 ASD 190 M: 34 F 2–6 years old86 OTD 63 M: 23 F 2–6 years old	133 Pts 81 M: 52 F	Experimental with a control group	Ethnicity of the patients: – Origin of the samples: Penn State Medical School, SUNY Center Socioeconomic level: – Diagnostic criteria: DSM-5, ADOS, MCHAT-R/F and Vineland Adaptive Behavior Scale	Standard method with TRIzol followed by a second round of purification using the RNeasy mini column	RNA-seq	DIANA-mirPath v3 and autism SFARI database	Hicks et al. [43]
	39 ASD 25 M: 14 F 3–7.5 years old 16 OTD 14 M: 2 F 3–6.5 years old	25 pts 11 M: 14 F 3.5–8 years old	Experimental with a control group	Ethnicity of the patients: Bosnioherzegovino Origin of the samples: Nongovernmental organization: EDUS-Education for All of Sarajevo Socioeconomic level: – Diagnostic criteria: EDUS Developmental Behavior Scales (EDUS-DBS) and CARS-II	mirVana kit	qRT–PCR	–	Sehovic et al. [8]
	77 pts 62 M: 15 F 7 years old (SD ± 1.5)	27 pts 29 M: 11 F 6.75 years old (SD ± 1.51)	Experimental with a control group	Ethnicity of the patients: –Origin of the samples: –Socioeconomic level: –Diagnostic criteria: ADOS, WISC-III and ADI-R	Qiagen miRNeasy Mini Kit (Qiagen, GmbH, Hilden, Germany)	NanoString platform and the nCounter Human v3 miRNA Expression Assay Kits (NanoString Technologies, Seattle, WA, USA)TaqMan Assays	DIANA-mirPath v.3 web server	Ragusa et al. [45]

*ASD* autism spectrum disorder, *CS* sample control, *ST* Tourette syndrome, *OTD* developmental disorders other than ASD, *PAC* primary auditory cortex, *STS* upper temporal sulcus, *ADOS* Autism Diagnostic Monitoring Program, *ADI-R* Autism Diagnostic Interview-Revised, *PMI* postmortem interval, *NICHD* Eunice Kennedy Shriver National Institute of Child Health and Human Development, *ICD-10* International Classification of Disorders, *CARS* Child Autism Rating Scale, *GARS* Gilliam Autism Rating Scale, *WAIS* Wechsler Intelligence Scale, *WISC-III* Wechsler Intelligence Scale for Children, 3rd edition, *SRS* Social Response Scale, RIN, M-CHAT-R/F Modified Early Detection Autism Questionnaire (for children aged 1–3 years old) with a Follow-up Interview, DAVID Database for Integrated Annotation, Visualization, and Discovery, (–) Not applicable due to data unavailability

**Table 2:** Deregulated microRNAs in different studies

Number of miRNAs studied	Differential expression criteria	miRNA with the highest differential expression	Deregulated miRNA		References
			Negatively deregulated miRNAs	Positively deregulated miRNAs	
1237	Pavlidis template matching analysis (PTM)	miR-182-AS FC = − 1.54 *P* = 1.44E−03	miR-182AS, miR-136, miR-518, miR-153-1, miR-520b, miR-455, miR-326, miR-199b, miR-16-2, miR-133b, miR-148b, miR-211, miR-132, miR-495, miR-190, miR-189, miR-367, miR-139, miR-219-	miR-185, miR-103, miR-107, miR-29b, miR-194, miR-524, miR-191, miR-376a-AS, miR-451, miR-23b, miR-195, miR-342, miR-23a, miR-25, miR-519c, miR-346, miR-205, miR-30c, miR-93, miR-186, miR-106b	Sarachana et al. [14]
708	FC 1.5 or higher in at least 12 of 24 pair comparisons of brothers	miR-199b-5p 1.81 FC *p* = 2.51E−05		miR-199b-5p, miR-548o miR-577, miR-486-3p miR-455-3p, miR-338-3p miR-199a-5p, miR-650 miR-486-5p, miR-125b miR-10a, miR-196a, miR-181c, miR-181a, miR-30a, miR-181b, miR-502-3p,	Seno et al. [22]
269 control vs ASD 267 brothers vs ASD	|log2 fold change| and *p* ≤ 0.05	miR-181a-5p − 1.1 FC *p* < 0.005 miR-320a − 1 FC *p* < 0.005	Controls vs ASD PC-5p-35875_59, mir-18b-p3, miR-451a_R-1, miR-181a-5p, miR-92a-2-5p_R + 1, PC-3p-16340_153, miR-20b-5p, let-7i-3p_R-2, miR-4437_L + 2, mir-5100-p3_1ss17TC, PC-3p-12325_216,miR-363-3p_R + 1, miR-10a-5p_R-1 Brothers vs ASD miR-451a_R-1, miR-99b-5p, PC-5p-8577_335, miR-320a, miR-96-5p_R-2, let-7e-5p, miR-4485-3p_L + 3R + 2, miR-4521_R + 3, miR-1270, miR-766-5p_R-1, miR-106a-5p, miR-125a-5p_R-1, miR-1246_L-1R + 1	Controls vs ASD miR-1271-5p, miR-151a-5p Brothers vs ASD miR-5701_1ss2TG, miR-150-5p	Frye et al. [29]
1104	FDR < 0. 05 and two-tailed *t* test for microRNA by real-time PCR	miR-338-5p 4.4 FC *p* = 5.47E−81	miR-211-5p, miR-34a-5p, miR-92b-3, miR-3960	miR-338-5p, miR-3168, miR-7-5p, miR-21-3p, miR-19a-3p, miR-19b-3p, miR-219-5p, miR-137, miR-146a-5p, miR-379-5p miR-494, miR-155-5p Por PCR in real time: miR-142-5p, miR-21-5p, miR-451a, miR-142-3p miR-144-3p	Mor et al. [31]
1733	*p* < 0.005 FC > 1.2	miR-297 − 1.24 FC *p* = 0.0012	miR-1, miR-297, miR-4742-3p	miR-4753-5p, miR-554-3p miR-4709-3p,	Ander et al. [15]
699	Mixed Effects Linear Model, FDR < 0.05	miR-3687 − 1.5 FC, FDR 1.5E−4	miR-1002, miR-1155, miR-619-5p, miR-3687	miR-21-3p, miR-2277-5p, miR-10a-5p, miR-424-5p, miR-199b-3p, miR-148a-9p	Wu et al. [2]
3	Fold change > 1.2 and *p* < 0.001 by the Mann–Whitney test	miR-146a	–	miR-146a	Nguyen et al. [32]
1	Non-parametric Mann–Whitney *U* test	miR-155p5 *p* < 0.0001	–	miR-155p5	Almehmadi et al. [30]
667	*p* < 0.01 according to Wilcoxon’s signed rank test and *p* < 0.05	miR-146a *p* < 0.001	miR-221, miR-654-5p, miR-656	miR-146a	Nguyen et al. [19]
125	Mann–Whitney *p* < 0.05	miR-572 *p* < 0.0001	miR-101-3p, miR-106b-5p,miR-130a-3p, miR-195-5p, miR-19b-3p, miR-27a-3p	miR-151a-3p, miR-433, miR-181b-5p, miR-328, miR-320a, miRR-572, miR-663a, miR-469,	Vasu et al. [40]
2578	Product range method *p* < 0.05 for micro-arrangements Wilcoxon range addition to test differences with *p* < 0.05 for qRT–PCR	miR-451a *p* = 4.58E−5	miR-451a, miR-16-5p, miR-574-3p, miR-7a-5p, miR-7d-5p, miR-7f-5p, miR-92a-3p, miR-3613-3p, miR-20a-5p, miR-3935, miR-4700-3p, miR-15b-5p, mir-15a-5p, miR-4436b-5p, miR-4665-5p, miR-19b-3p, miR-195-5p, miR-103a-3p, miR-1228-3p, miR-940,	miR-1273c, miR-4299, miR-5739, miR-6086, miR-494, miR-4270, miR-642a-3p, miR-4516, miR-4436a, miR-1246, miR-575 miR-4721, miR-483-5p, miR-1249, miR-4443, miR-921, miR-34b-3p, miR-6125, miR-4669, miR-34c-3p, miR-4728-5p, miR-564, miR-574-5p, miR-4788	Huang et al. [34]
42	FC > 0.5	miR-619-5p FC = 2.983	Preliminary studies: miR-589-3p, miR-6849-3p, miR-15a-5p, miR-183-5p, miR-3674, miR-96-5p, miR-3687, miR-6799-3p, miR-587-3p, miR-504-5p, miR-576-5p, miR-486-3p, miR-3909, miR-29c-5p, miR-301a-3p, miR-3064-5p, miR-145-5p, miR-193b-3p, miR-487b-3p, miR- 664b-3p, miR-20b-3p, miR-671-3p, miR-199a-5p Confirmation by qRT–PCR: miR-3135a, miR-328-3p, miR-197-5p, miR-500a-5p and miR-424-5p	Preliminary studies: miR-4489, miR -8052, miR-106b-5p, miR-142-3p, miR-3620-3p, miR-374b-5p, miR-18b-3p, miR-210-5p Confirmation by qRT–PCR: miR-365a-3p, miR-619-5p and miR-664a-3p	Kichukova et al. [36]
754	Test of two classes not paired with FDR < 0.15 for micro-arrangements One-way ANOVA *p* ≤ 0.05 for qRT–PCR	miR-140-3p *p* < 0.0001 FC = 1.42		miR-140-3p	Cirnigliaro et al. [33]
702	Fisher’s exact test and Spearman’s test *p* < 0.05	miR-342 FC = 2.03	miR-30e, miR-30c-1, miR-101-1, miR-186, miR-197, miR-9-1, miR-199a-2, miR-181b-1, miR-181a-1, miR-29c, miR-29b-2, miR-664a, miR-4433b, miR-9-2 miR-128-1, miR-128-2, miR-425, miR-191, miR-15b, miR-16-2, miR-28, miR-582, miR-143 miR-22 miR-145, miR-378a, miR-103a-1, miR-340, miR-30c-2, miR-339, miR-148a, miR-25, miR-93, miR-106b, miR-335, miR-29a, miR-29b-1, miR-671, miR-320a, miR-486, miR-486-2, miR-30b, miR-151a, miR-101-2, miR-7-1, miR-23b, miR-27b, miR-24-1, miR-181a-2, miR-181b-2 miR-505 miR-199b, miR-126, miR-107, miR-146b, miR-130a, miR-6503, miR-139, miR-326, miR-148b, miR-26a-2, miR-331, miR-16-1, miR-15a, miR-17, miR-19a, miR-19b-1, miR-92a-1, miR-625, miR-342, miR-345, miR-411, miR-329-1, miR-329-2, miR-494, miR-543, miR-495, miR-376c, miR-376b, miR-487b, miR-134, miR-485, miR-496, miR-409, miR-9-3, miR-484, miR-424 miR-324, miR-423, miR-142, miR-454, miR-301a, miR-21, miR-338, miR-7-3, miR-199a-1, miR-24-2, miR-27a, miR-23a, miR-150, miR-99b, miR-125a, miR-103a-2, miR-133a-2, miR-99a, mir-155, miR-185, miR-130b, mir-221, miR-222, miR-660, miR-223, miR-421, miR-374b, miR-652, miR-766, miR-92a-2, miR-19b-2, miR-20b	miR-1248, miR-4485, miR-7-2, miR-365a, miR-365b, miR-769, miR-98, miR-545	Jyonouchi et al. [35]
26	Student’s *t* test, Waller–Duncan test and Tukey HSD test with SPSS 17	–	miR-19b-1-5p, miR-27a-3p, miR-193a-5p	miR-34c-5p, miR-145-5p, miR-199a-5p	Vaccaro et al. [39]
43	FC*, p* < 0.005 Student’s *t* test One-way analysis of variance	miR-486-3p *p* < 0.001 FC = 2.566	Microarrays miR-6785-3p, miR-6819-3p	Microarrays miR-6802-5p, miR-6808-5p, miR-6829-5p, miR-6865-5p, miR-5100, miR-6086, miR-197-3p, miR-106b-5p, miR-1185-1-3p, miR-1249-5p, miR-140-3p, miR-1471, miR-188-5p hsa-miR-19a-3p, miR-24-3p, miR-296-5p, miR-30d-5p, miR-3141, miR-3196, miR-342-3p, miR-3648, miR-3667-5p, miR-3945, miR-4429, miR-4472, miR-4532, miR-4655-3p, miR-4728-5p, miR-4745-5p, miR-4778-5p, miR-4800-5p, miR-5006-5p, miR-5195-3p, miR-5585-3p, miR-6090 miR-642b-3p, miR-6752-3p, miR-6768-5p, miR-6785-5p Confirmation by qRT–PCR: miR-486-3p, miR-557	Yu et al. [41]
3	Analysis of variance (ANOVA) Student’s *t* test *p* < 0.05	–	miR-3135a, miR-328-3p	–	Nt et al. [19]
2549	*p* < 0.01 FC > 1.5	miR-6126 *p* = 2.00E−05 FDR = 7.58E−03	miR-6126, miR-6780a-5p, miR-1227-5p, miR-3156-5p, miR-4716-3p, miR-144-3p, miR-3127-5p, miR-5581-5p, miR-6756-5p, miR-6767-5p, miR-7977, miR-4486, miR-6734-5p, miR-4653-3p, miR-6085, miR-874-3p	miR-328-3p, miR-4515	Nakata et al. [37]
27	Mann–Whitney two-tailed *p* < 0.05	miR193a-5p	miR-193a-5p, miR-576-3p, miR-382-5p, miR-134-5p, miR-7-5p, miR-379-5p, miR-27a-5p, miR378a-3p, miR-574-3p, miR-223-5p, miR-433-3p, miR-3614-5p, mir-872-3p, miR-103a-3p	miR-206, miR-4732-5p, miR-184	Jyonouchi et al. [24]
280	*p* < 0.05	miR-150-5p FC: − 8.68	miR-19a-3p, miR-361-5p, miR-3613-3p, miR-150-5p, miR-126-3p, miR-499a-5p	–	Ozkul et al. [38]
3	Kolmogorov–Smirnov test, Independent *t* test, Mann/Whitney *U* test, Kruskal–Wallis test, and ROC curve *p* < 0.01	miR-23a FC = 1.99 *P* = 0.18	miR-181b	miR-23a, miR-181b	Atwan et al. [7]
1	Statistical Package for Social Sciences version 20 (SPSS software, Chicago, USA) Chi-square tests Statistical significance was considered at *p* < 0.05, while *p* < 0.01 was considered highly statistically significant	miR-106a*p* = 0.000	–	miR-106a	Zamil et al. [42]
246	Mann–Whitney FDR < 0.15 *p* < 0.05	miR-628-5p difference in Z 1,13, FDR 0.027 *p* < 0.0001	miR-23a-3p, miR-27a-3p, miR-30e-5p, miR-32-5p,	miR-7-5p, miR-28-5p, miR-127-3p, miR-140-3p, miR-191-5p, miR-628-5p, miR-3529-3p, miR-2467-5, miR-218-50, miR-335-3p	Hicks et al. [44]
11	Characteristic selection algorithm	–	–	miR-92a-3p, miR-146b, miR-3916, miR-146b-5p, miR-378a-3p, miR-361-5p miR-125a-5p, miR-410 miR-106a-5p, miR-146a, miR-10a	Hicks et al. [16]
527	Non-parametric Kruskal–Wallis test and a partial least-squares discriminant analysis (PLS-DA) FDR < 0.05 PLS-DA ≥ 2.0	miR-28-3p χ2 = 34 FDR = 1.62E-5	miR-148a-5p, miR-151-3p miR-125b-2-3p, miR-7706, mir-28-3p	miR-28-3p, miR-665, miR-4705, miR-620, miR-1277-5p	Hicks et al. [43]
14	Testing Grubbs through the Xlstat add-on with the program Microsoft Office Excel Z > 1.5Mann–Whitney test	miR-32-5p	miR-23a-3p, miR-32-5p, miR-140-3p, miR-628-5p	miR-7-5p, miR-2467-5p	Sehovic et al. [8]
800	SAM (Significance of Microarrays Analysis) Fold change (FC) values The Mann–Whitney *U* test *p* < 0.05	miR-451a FC = − 3.58 *P* < 0.0001	miR-16-5p, miR-205-5p, miR-451a, let-7b-5p	miR-29a-3p, miR-141-3p, miR-146a-5p, miR-200a-3p, miR-200b-3p, miR-4454, miR-7975	Ragusa et al. [45]

*FC* fold change, *FDR* Rogers discriminant function, One-way ANOVA single-factor analysis of variance

Although 218 miRNAs were identified across the 16 studies included in the meta-analysis, only one (miR-451) was associated with a clinical manifestation of ASD in more than one study. Two studies [31, 45] reported that miR-451 is associated with impaired social interaction, one study [43] reported that miR-106 family is associated with repetitive behaviors, one study [41] reported that miR-486-3p is associated with intelligence, one study [33] reported that miR-140-3p is associated with memory and learning, and no studies reported that any miRNA was associated with language impairment.

Regarding diagnoses, all studies used certain validated instruments to diagnose autism, including the ADOS, ADI-R-R, DSM, M-CHAT, and CARS. Two studies concluded that miRNAs are associated with severity. The expression of miR-6126 was significantly negative correlated with severity of the Social Response Scale (SRS) in adults with high functioning ASD [37] and miR-106a showed a positive correlation with autism severity evaluated by Childhood Autism Rating Scale (CARS) [42] in children aged under 14.

**Fig. 2:**
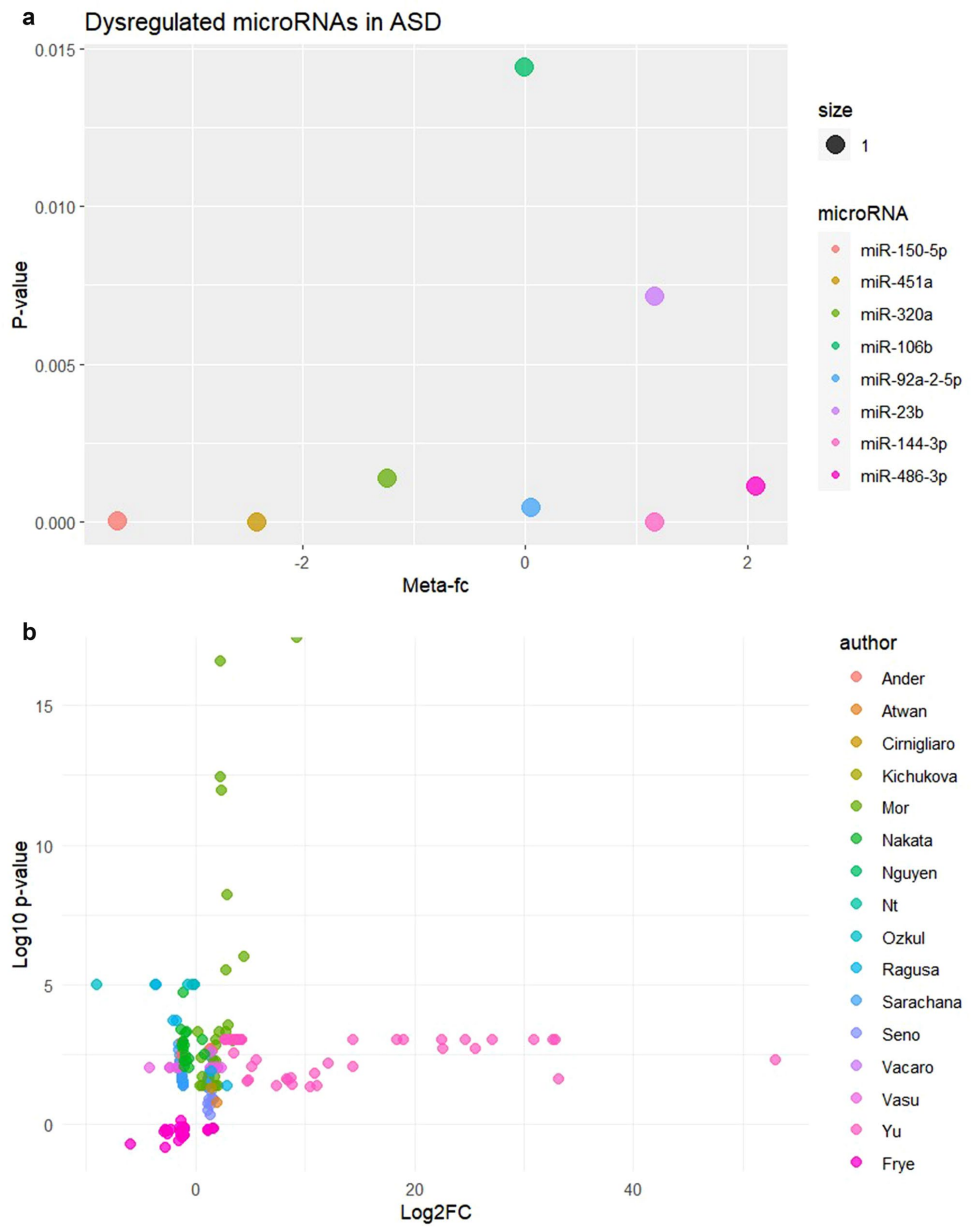
**a** Combined results of the fold change for the expression of eight miRNAs. **b** 16 studies included in the meta-analysis and the fold change of the expression in every miRNA

**Fig. 3:**
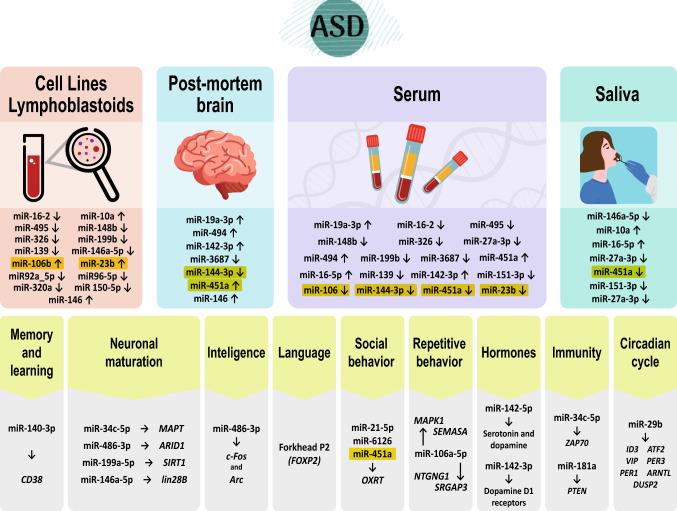
Target genes of the analyzed miRNAs and miRNAs related to different clinical manifestations of ASD

For our meta-analytic approach, we included 16 studies and eight miRNAs (451-a, 144-3p, 23b, 106b, 150-5p, 320a, 92a-2-5p, and 486-3p) whose data were reported in more than one study. Since no studies reported the confidence interval for fc, we were unable to conduct all planned analyses. We used a combined approach to summarize the fold changes of the miRNAs in different studies according to the mean values. In addition, we summarized the *p* values for differential miRNA expression using the Fisher method. The combined results for the eight miRNAs are shown in Table 3 and Fig. 2a, b shows the 16 studies included in the meta-analysis and the fold change of the expression in every miRNA. Each circle represents a miRNA. 218 miRNAs were identified across 16 studies but only 8 miRNAs were repeated in more than one study. Figure 3 summarizes all the target genes of the analyzed miRNAs and shows the miRNAs related to different clinical manifestations of ASD. Finally, Table 4 shows target genes of the analyzed miRNAs; and Fig. S1. Quality assessment of individual studies is done using QUADAS tool.

**Table 3:** Combined result of log2-fc from the meta-analysis

microRNA	Meta-fc	*p* value
miR-451a	− 2.4175	6.47E−25
miR-150-5p	− 3.6757	5.12E−05
miR-486-3p	2.0780	1.16E−03
miR-144-3p	1.1675	1.93E−05
miR-320a	− 1.2500	1.39E−03
miR-23b	1.1650	7.17E−03
miR-92a-2-5p	0.0500	4.91E−04
miR-106b	− 0.0150	1.44E−02

*FC* fold change

**Table 4:** Genes and miRNAs

miRNA	*Gen*	Related to	References
miR-29b	*ID3*	Circadian rhythm signaling	Sarachana et al. [14]
	*COL6A2*	Motor impairments and muscular disorders	
	*CLIC1*	Stabilization of cell membrane potential	
	*ARPC5*	Cell migration	
	*KIF26B*	Cell signaling	
	*ARNTL2*	Circadian rhythm signaling	
	*BMAL1*		
	*ATF2*		
	*DUSP2*		
	*PER1 y PER3*		
	*VIP*		
miR-219-5p	*PLK2*	Regulation of cell cycle and homeostatic plasticity of hippocampal neurons	
miR-139-5p	*PDE4DIP*	Brain size	
miR-199b-5p	*HES1*	NOTCH signaling network and Central Nervous System development	Ghahramani Seno et al. [22]
	*SET*	Cell growth and differentiation	
miR-486	*SFRS3*	Memory formation	
	*PTEN*	Genomic stability, cell survival, migration, proliferation and metabolism	
miR-181	*ATG5*	Autophagy regulation	Frye et al. [29]
	*AKT3*	PTEN/Akt/TGF-β1 Regulation	
miR-320a		PI3K-AKT-mTOR pathway regulation	
miR-21-5p	*OXTR*	Social behavior	Mor et al. [31]
miR-451a			
MiR-142-5p	*SIRT1*	Inflammatory signaling in response to environmental stress, development and placental cell survival	
miR-146a, miR146b	–	AMPA receptor endocytosis and glial proliferation via Notch signaling	Ander et al. [15]
miR-4753-5p	–	Axon guidance, neurotrophin and GnRH signaling, gap junctions and synaptic vesicle cycle	
miR-21-3p	*PAFAH1B1/LIS1*	Cell migration	Wu et al. [2]
	*DLGAP1*	Scaffold protein that binds to the protein product of *SHANK3*, an ASD risk gene	
	*TP2B1/PMCA1*	Cell migration	
	*NEEP21*	Synaptic transmission	
	*SV2B*	Synaptic transmission	
miR-146a	*CHEK1, BRCA1, BRCA2, CCNA2, TIMELESS, CDCA5, E2F2, KIF18A, DCX, PAK3, IRS1, GAD1, EPB41, MYBL1, IQGAP3*	Cell cycle and neuronal differentiation	Nguyen et al. [32]
	*SLC17A8*	Synaptic transmission	
	*LIN28B*	Neuronal proliferation	
	*CDKN1A, CDKN3 y CDK1*	Cell cycle y el equilibrio entre el mantenimiento del progenitor y neuronal differentiation	
miR-155p	*SOCS-1*	Cytokine signaling and inflammatory response	Almehmadi et al. [30]
miR-146a	*MAP1B*	Synaptic transmission	Nguyen et al. [18]
miR-146a miR-221	*GRIA3*	Synaptic transmission	
	*KCNK2*	Synaptic transmission and neuronal migration	
miR-656	*MAP2*	Neuritogenesis and neuron morphogenesis during neuron development	
miR-130a-3p, miR-19b-3p, miR-320a, miR181b-5p y miR-572	–	TGF-beta signaling, MAPK signaling, adherens junction and focal adhesion, regulation of actin cytoskeleton, oxidative phosphorylation, hedgehog, mTOR and Wnt signaling	Mundalil Vasu et al. [40]
miR-34b		Central Nervous System neuron development	Huang et al. [34]
miR-103a-3p	*BTRC*	Circadian rhythm signaling. Ubiquitin-mediated proteolysis. Wnt signaling	
	*BDNF*	Neural development, neuronal proliferation and differentiation, synaptic transmission, neuronal survival, memory and cognition	
	–	Central Nervous System development, neuritogeneis and neuron morphogenesis, synapstic transmission. Skeletal muscle tissue and organ development	
miR-let-7a	*TLX*	Neuronal proliferation	
miR-1228-3p	*HGF*	Paracrine cellular growth, motility and morphogenic signaling	
miR-328-3p y miR-619-5p	*CACNA1C*	Calcium channels: regulation of electrical activity in cells	Kichukova et al. [36]
	*CACNB1*	Calcium channels: regulation of electrical activity in cells	
	*DICER 1*	Epigenetic processes	
miR-424-5p	*RNASEN*	Epigenetic processes	
miR-140-3p	*CD38* *NRIP1*	Brain development, synaptic transmission, social behavior, memory and cognition. Circadian rhythm signaling. Response to estradiol and steroid hormones, reproductive system development, and development of primary sexual characteristics	Cirnigliaro et al. [33]
miR-181a	*PTEN*	Neuroinflammation	Jyonouchi et al. [35]
miR-93	*PTEN y PHLPP2*	Neuroinflammation	
miR-34c-5p	*NANOG NOTCH1, SOX2 SRSF2, NOTCH4* *E2F3, MYCN MYC, CCNE2* *BCL2*	Epigenetic processes	Vaccaro et al. [39]
	*CCNE2, BCL2,*	Cell cycle	
	*ZAP70, ULBP2, CDK4, CAV1*	Neuroinflammation	
	*MAPT*	Cytoskeletal modulation in neurons	
	*UNG*	DNA repair	
miR-145	*ERS1, POU5F1, C11orf9, PARP8, SOX2, HOX9, STAT1, KLF4, KLF5, NEDD9, DDX17, EIF4E, CBFB, HDAC2*	Epigenetic processes	
	*CDKN1A, CDK4, MYC, PPM1D, KRT7*	Cell cycle	
	*IFNB1, TIRAP, SOCS7, ADAM17*	Immunological response	
	*IGF1R, IRS1, IRS2*	Insulin metabolism	
	*ZAP70, ILK, MYO6, FSCN1, ROBO2, CDH2, TMOD3, SRGAP1PAK4*	Cytoskeletal modulation and cell migration	
miR-199a	*SIRT1, SOX9, MED6,* *SMARCA2, CCNL1, JUNB, HIF1A, ETS2, MECP2*	Epigenetic processes	
	*AV1, SMAD4, ALOX5AP, CD44, IKBKB*	Epigenetic processes	
	*ERBB2*	Cytoskeletal modulation and cell migration	
	*UNG*	DNA repair	
	*SULT1E1*	Estrogen metabolism	
miR-19b	*CCND1*	Cell cycle	
	*ITGB8, KDR*	Cytoskeletal modulation	
miR-27a	*SP4, SP3, WDR77, RUNX1, MYT1, SP1, FOXO1, PAX3, NFE2L2, HIPK2, ZBTB10*	Epigenetic processes	
	*PHB, WEE1*	Cell cycle	
miR-193a	*mTOR*	Major regulator of metabolism and physiology with important roles in the function of tissues including the brain	
	*TP73*	Cell cycle	
miR-486-3p	*ARID1B*	Neuronal differentiation and brain development	Yu et al. [41]
miR-328-3p miR-3135a	*APP*	Synaptic transmission	Nt et al. [19]
	*SLC8A1*	Membrane repolarization in neurons	
	*BACE1*	Proteolytic processing of the Amyloid Precursor Protein (APP)	
miR-6126	*ANK3, CACNA2D1, NRXN3, PCDH9*	Candidate ASD genes	Nakata et al. [37]
	–	Axon guidance. Oxytocin signaling pathways	
–	–	Cell proliferation and differentiation, synaptic formation and plasticity (neurotrophin signaling and axon guidance)	Jyonouchi et al. [24]
miR-19a-3p, miR-361-5p, miR-3613-3p, miR-150-5p, miR-126-3p y miR-499a- 5p	*CC2D1A*	Embryonic development. Synaptic transmission of serotonin	Ozkul et al. [38]
miR-181b-5p, miR-23a-3p	*BCL-2*	Inflammatory response and apoptosis	Atwan et al. [7]
miR-106a	*ADARB1*	Post-transcriptional modification	Zamil et al. [42]
–	*FOXP2*	Speech and language development	Hicks et al. [44]
–	–	Chromatin remodeling. Post-transcriptional modification. Synaptic transmission	Hicks et al. [16]
miR-410	*FMR1*	Synaptic transmission	
miR-10a	*PTEN*	Neuroinflammation. Mitochondrial dysfunction	
	*BDNF*	Neural development, neuronal proliferation and differentiation, synaptic transmission, neuronal survival, memory and cognition	
miR-92a	*TSC1*	Cell growth and size	
miR-106a	*SCN2A*	Sodium channels: initiation and conduction of action potentials	
mir-361	*GSTO2*	Metabolism of xenobiotics and carcinogens	
miR-125a	*GSTM2*	Metabolism of xenobiotics and carcinogens	
miR-148a-5p	*SRGAP3*	Axon guidance. Candidate ASD gene	Hicks et al. [43]
	*SLIT3*	Axon guidance by interacting with the product of *ROBO1,* an ASD risk gene	
miR-944a	*ROBO1*	Axon guidance and adherens junction	
miR-106a-5p	*SEMA5A, NTNG1, SRGAP3 y MAPK1*	Candidate ASD gene	
miR-23a-3p	–	Cell proliferation and differentiation	Sehovic et al. [8]
miR-27a-3p			
miR-141-3p	*PTEN*	Neuroinflammation. Mitochondrial dysfunction	Ragusa et al. [45]
	*MAP4K4*	Cell migration, proliferation and adhesion	

## Discussion

This review summarizes findings across 27 trials conducted in humans. To our knowledge, this is the first systematic review and meta-analysis of dysregulated miRNAs in ASD, their associations with ASD clinical manifestations, and miRNA measurement in biofluids from individuals with ASD. (i) The most frequently dysregulated miRNAs in patients with ASD were miR-451a, miR-144-3p, miR-23b, miR-106b, 150-5p, 320a, 92a-2-5p, and 486-3p, (ii) miR-451 is one of the most frequently dysregulated miRNAs and is the only associated with impaired social interaction in more than one study. (iii) miR-451 has also been isolated in saliva and may be the most promising biofluid for miRNA measurement. (iv) miRNA 106 family is also one of the most frequently dysregulated miRNAs and is associated with repetitive behaviors in one of the studies. (v) miRNAs are associated with genes related to ASD.

Although behavioral signs of ASD are present in many cases by the age of 18 months, ASD is not typically diagnosed before 3 to 4 years of age. To date, the only means to diagnose ASD is by observing children’s development through neurodevelopmental evaluations. No biological marker allows detection of the disease from birth; therefore, one of the current challenges for researchers is to determine whether miRNAs can truly be used as biomarkers to facilitate ASD diagnosis. We found that although research on miRNAs for the diagnosis of ASD started 12 years ago, at present, no pattern of miRNAs specific to ASD clinical levels of severity has been identified, and specific sets of miRNA do not reveal a pattern, rather than big data emergent assessment across all miRNA. Only one paper [43] 2020 suggests that there is a specific pattern. MiRNA measurement in different body fluids, such as blood or saliva, may be useful for comparing the levels of a specific miRNA to control levels, which can be applied to the search for a biological marker of ASD.

On the other hand, although transcriptomic or genetic analysis can be performed from birth, ASD is a disease with a heterogeneous genetic component, and no specific gene is universally affected in the entire population with ASD; therefore, performing a genetic or transcriptomic analysis is not feasible. However, as miRNAs are epigenetic modulators, the study of epigenetics can help identify one or more specific miRNAs that can be used as biomarkers.

An advantage of circulating miRNAs is that they are highly stable in the presence of RNases, resist pH changes, remain viable after prolonged storage, and resist freeze–thaw cycles [21]. Notably, miRNAs expressed in brain tissues are functionally or physiologically related to the physiopathology of ASD. Additionally, miRNAs in blood or saliva can reflect brain miRNA levels [36] and may be specific biomarkers for the diagnosis of ASD.

### Frequently dysregulated miRNAs

The most frequently dysregulated miRNAs include miR-451a, miR-144-3p, miR-23b, and miR-106b (see Table 4). Additionally, miRNAs, including miR-16-2, miR-16-5p, miR-495, miR-148b, miR-326, miR-139, miR-199b, miR19a-3p, miR-494, miR-142-3p, miR-3687, and miR-27a-3p, are differentially expressed in various tissues and body fluids in patients with ASD. Among the miRNAs that were reported as dysregulated but excluded from the meta-analyses because there was not enough data available across studies are miR-140-3p, miR-34c-5p, miR-483-3p, miR-199a-5p, miR-142-5p, miR-142-3p, miR-21-5p, miR-6126 and miR-106a-5p, miR-146a, miR-193a, miR-181a, miR-155-5p, miR-483-3p, miR-34b-3p, miR-29b, miR141-3p, let7b-5p, and miR165p, which also induce the regulation of genes related to ASD.

Although several studies with very small populations identified different dysregulated miRNAs in isolation, other studies with large and representative samples [43] have detected alterations in several miRNAs whose dysregulation appears concurrently and have established an algorithm for four miRNAs (miR-28-3p, miR-151-a-3p, miR-148a-5p, miR-125b-2-3p) to differentiate children with ASD from healthy individuals and ASD from other developmental disorders. MiR-125b-2-3p and miR-151a-3p were found to be associated with ASD features in the ADOS assessment. This algorithm demonstrated a sensitivity of 89% and a specificity of 32%.

### miRNA and the genetic load in ASD-associated syndromes

Dysregulation of miRNAs between families is also evident. Ozkul et al. [38] identified a group of miRNAs (miR-19a-3p, miR-361-5p, miR-3613-3p, miR-150-5p, miR-126-3p and miR-499a-5) that were profoundly decreased in patients with ASD and moderately decreased in their relatives who did not develop the disease compared to nongenetically related healthy controls, implying a potential heritability pattern in which the most serious phenotype of ASD has the lowest levels of miRNAs, as observed in children with ASD, and another phenotype in which the disease does not emerge and the levels of miRNAs are moderately low, as observed in the parents and siblings of patients with ASD.

Rett syndrome is a monogenic disorder linked to the X chromosome and is caused by mutations in the *MECP2* gene, which prevent its binding to methylated DNA, thus repressing gene translation and consequently the development of autistic behavior. MiR-199 exerts epigenetic regulation on *MECP2* [39]. In addition, miR-132 also targets this gene [40], corroborating that miRNAs can play an important role in gene regulation. Peripheral blood studies show that miR-140-3p is differentially upregulated in patients with ASD compared to controls and in patients suffering from Tourette syndrome and ASD. Therefore, miR-140-3p may be a candidate biomarker for the differential diagnosis of ASD [33]. The *FMR1* gene is widely expressed in neurons and is regulated by 14 miRNAs that are dysregulated in ASD [44]. This gene is altered in fragile X syndrome, which causes intellectual disability, and 40% of patients with this disease meet the diagnostic criteria for ASD.

### Hormones and epigenetic mechanisms

miRNAs, as epigenetic modulators, affect the protein levels of the target mRNAs without modifying the gene sequences. Moreover, miRNA can also be regulated by epigenetic modifications [31, 47]. Therefore, epigenetics could play a role in dysregulation of miRNAs. Previous studies have found a dysregulation in methylation and acetylation patterns of miRNAs in the brain of humans with ASD. Mor et al. [31] showed that dysregulated miRNAs target biological pathways and specific genes, modifying their expression levels, that are highly relevant to the biology of autism. An interesting example is the oxytocin receptor (OXTR) gene, which codified for the receptor for the hormone and neurotransmitter oxytocin. OXTR acts as a vascular regulator or an inducer of uterine contractions during parturition. However, in the central nervous system [48–50], OXTR is associated with roles in social, cognitive, and emotional behavior. Regarding these functions, perturbations in OXTR have been implicated in subpopulations of individuals with ASD, including Asperger’s syndrome.

miR-142 has important roles in the dopaminergic and monoamine pathways in the brain. Moreover, this miRNA can also target OXTR gene and modulate their expression. Mor et al. [31] evidenced that epigenetics plays a role in dysregulation of miR-142 in the brain of ASD patients. Particularly, they found a hypomethylation in five CpG sites in the promoter region of the gene coding for miR-142, which correlates with elevated levels of miR-142, resulting in effects on OXTR gene expression that may favor the pathogenesis of ASD.

As epigenetic modulator, miR-142-5p negatively regulates the transcription of monoamine oxidase, thus influencing the metabolism of neurotransmitters such as serotonin and dopamine. Moreover, miR-142-5p targets and decreases the translation of dopamine D1 receptors. Therefore, this miRNA has important functions in the dopaminergic and monoamine pathways of the brain that are strongly related to ASD. In this line, miR-142-5p was found to be upregulated in patients with ASD and may be involved in the degradation of HDAC2 mRNA, generating alterations in cell differentiation and proliferation [31].

### Neuronal maturation and ASD

ASD is characterized by its association with neuronal maturation, neuronal plasticity, neurogenesis, and neuronal functions [41]. A gene associated with stabilization of the neuronal cytoskeleton (*MAPT*) is a target of miR-34c-5p, which inhibits its expression and generates effects on neuronal maturation [39].

The studies by Nguyen et al. [32] show that miR-146a overexpression in patients with ASD induces negative regulation of the *LIN28B* gene, which encodes a protein whose function is to maintain neural progenitors in an early stage of neuroblast proliferation. Additionally, miR-146a participates in negative regulation of *CDKN1A, CDKN3,* and *CDK1*, which encode proteins responsible for controlling the duration of the G1 phase during the cell cycle and the balance between the maintenance of progenitor cells and the emergence of differentiated neurons, which may be involved in the pathophysiology of ASD.

### Social interaction and miRNAs

One of the functions of oxytocin is activation of the OXRT receptor, which, in the central nervous system, is mainly related to the biological signals of impaired social interaction. Overexpression of miR-21-5p and miR-451a is correlated with elevated *OXRT* mRNA expression, and, in turn, miR21-5p expression is correlated with low levels of the OXRT receptor. This hypothesis suggests that miR-21-5p inhibits translation of *OXRT* [31]. In addition, miR-6126 is closely related to oxytocin signaling pathways [37]. Changes induced in the *OXRT* pathway either by miRNA or by single-nucleotide polymorphisms have been associated with ASD, particularly in patients with Asperger’s syndrome. The deregulation of miR-21-5p, miR-451a and miR-6126 is related to alterations in social interactions, which are characteristic of ASD [31].

### Repetitive behaviors

Ten miRNAs [43] are related to the manifestation of restricted and repetitive behaviors in patients with ASD, highlighting miR-106a-5p, which is responsible for the regulation of genes that are candidates for ASD (*SEMA5A, NTNG1, SRGAP3, and MAPK1*). Therefore, the dysregulation of miR-106a-5p can target transcripts that are related to brain development and lead to restricted and repetitive behaviors.

### MicroRNAs involved in immunity in ASD

Neuroimmune interactions can originate during embryogenesis. Accordingly, children with ASD present altered immune responses, including altered cytokine TH1/TH2 profiles, low NK activity, chronic neuroinflammation generated by glutamatergic excitotoxicity and decreased GABAergic signals, and imbalances in serum immunoglobulins.

The excitatory–inhibitory imbalance hypothesis postulates dysregulation of the GABA and glutamate neurotransmission is associated with deficits in individuals with ASD [51]. According to this theory, the imbalanced neurotransmission results in increased noise and hyperexcitability in the cerebral cortex of these patients. Later, Blaylock and Strunecka postulated a link between perturbed glutamatergic neurotransmission and pro-inflammatory changes in the ASD brain. The author introduced the term “immunoexcitotoxicity’ to describe neuronal injury hypothesized to result from microglial activation in ASD brain, since chronic activation of microglia derives in a predominant neurotoxic effect on the brain, with excitotoxic levels of glutamate being secreted [52].

In the peripheral blood of patients with ASD, miR-34c-5p, which targets *ZAP70,* gene implicated in lymphocyte activation and NK acyivity, is profoundly dysregulated, reflecting a decrease in the CD4 + population and an imbalance of the Th1/Th2 subsets toward Th2 [39].

The cellular and differentiation functions of macrophages and monocytes, lymphocytes, and NK are partly regulated by miRNAs; for example, miR-181a was identified in patients with ASD and decreased IL-B and high IL-10 profiles in the studies performed by Jyonouchi et al. [24, 35]. These studies showed a decrease in this miRNA. MiR-181a was also determined to directly regulate the inflammatory response mediated by macrophages and monocytes through negative regulation of pro-inflammatory cytokines and suppression of PTEN-related signaling pathways, thus affecting regulatory T-cell differentiation as well as mitochondrial functions. Therefore, IL-B and IL-10 profiles together with miRNA levels may be biomarkers for immune-mediated inflammation in ASD. Moreover, recent research [29] found that all miR-181 family members, TNFa levels and NK profiles are dysregulated in ASD.

### MicroRNAs, intelligence, learning, language, and memory in ASD

Yu et al. [41] demonstrated that miR-483-3p is dysregulated in ASD patients, leading to changes in the expression of c-Fos and Arc, which have an effect on dendritic and synaptic development, and thus contributing to the pathology of ASD, specifically impairments in intelligence and behavior. The study by Hicks et al. [44], which involved 14 miRNAs as possible biomarkers of ASD, shows that these miRNAs are expressed in different areas of the brain at different childhood ages. Analysis of the genes targeted by these miRNAs highlights the Forkhead P2 box protein (FOXP2), which has been heavily implicated in speech and language disorders.

Mutations in this gene result in a very characteristic verbal apraxia of fragile X Syndrome. While verbal apraxia is typically found in fragile X Syndrome, this alteration is also a hallmark of ASD. Interestingly, about 40% of the children with Fragile X Syndrome meet the criteria for ASD [44]. The regulatory network mediated by miR-140-3p plays a role in the CNS, and its dysregulation leads to alteration of *CD38* gene*,* which is involved in learning, postnatal glial development, and hippocampal-dependent memory [33]. Mir-34b-3p, which is also dysregulated in patients with ASD, is related to neuronal development and long-term memory [34]. Therefore, these miRNAs, which are dysregulated in patients with ASD and physiologically related to the disease, are good biomarker candidates for ASD.

### Biofluids for microRNA measurement

Expression of most miRNAs takes place within the cells themselves in every cerebral region. Nonetheless, several miRNAs, known as circulating miRNAs, have been found in human biological fluids like saliva, urine, blood, or cerebrospinal fluid [53]. Up to today's date, the known ways of secretion of circulating miRNAs are the following ones: (1) damaged cells, due to apoptosis or necrosis, which produce a passive secretion; (2) usage of extracellular vesicles to create an active secretion; (3) usage of RNA-binding protein-dependent pathways to generate an active secretion [54]. The most promising and advantageous biofluid in ASD, regarding the number of detected circulating miRNAs, is the saliva above the others.

It is available on demand and is quickly renewed in most adults, adolescents, and healthy children. It is also inexpensive to obtain, and collection is fast [55]. Initially, lymphoblastoid cells and cerebral cortex tissues were used for miRNA measurement. However, lymphoblastoid cells are not an ideal sample for determining the levels of miRNAs. The physiological relevance of miRNA expression in lymphoblastoid cell cultures raises methodological concerns [43] due to controversies regarding whether they are relevant indicators of neuronal tissue. Nevertheless, an unlimited amount of miRNA can be collected from these tissues [22]. In patients with ASD, sample collection can be complicated, since they can develop irritability with body contact and with some disturbances that arise in children’s daily lives [56]. Blood extraction is complicated, and preanalytical phase errors such as improper sample collection could occur. In addition, blood extraction is painful, requires specific personnel, and can generate anxiety and physical malaise [55]. Postmortem cerebral cortex tissues may be an ideal sample for studying and measuring miRNA considering that 70% of miRNAs are synthesized in the brain. However, postmortem samples of the cerebral cortex are generally taken from adults, which limits related analyses, since miRNA levels may vary considerably between childhood and adulthood, and the goal in the future is to develop a technique that allows early diagnosis [57].

## Strengths and limitations

Some limitations should be noted, first, we were not able to accurately analyze all microRNAs, since there was not enough data available across studies. In particular, only 16 of 27 studies in the review could be included in the meta-analysis as the others had incomplete data for confidence intervals for the fc of every miRNA. Second, the studies used different metrics to statistically analyze whether a certain microRNA is increased or decreased in ASD patients compared to the negative control, so many studies that showed very promising results had to be excluded as we did not find a common variable that could be analyzed; and third, although 16 studies were included in the meta-analysis, not all studies choose the same microRNA; as a result, some studies that reported FC value had to be excluded as there was no other study that analyzed the same microRNA for fair comparison. Finally, no raw data were available in most studies.

We believe the main strength of our study is the disclosing of concrete clinical implications that may contribute to a better knowledge on the relationship between miRNAs and ASD. Of particular importance is the finding that the variability in both the miRNAs chosen and the metrics used for analyses in most studies to date highlights the need to establish protocols to compare consistent results. It is noteworthy that 218 miRNAs were identified across the 16 meta-analyzed studies, 8 studies were comparable, and only one was able to replicate the results in terms of clinical manifestations.

## Conclusion

The most frequently dysregulated miRNAs across the analyzed studies were miR-451a, miR-144-3p, miR-23b, miR-106b, miR150-5p, miR320a, miR92a-2-5p, and miR486-3p. Therefore, all these miRNAs can be considered candidates for ASD biomarkers. Among the most dysregulated miRNAs in individuals with ASD, miR-451a is the most relevant to clinical practice and is associated with impaired social interaction in patients with ASD. Saliva may be the optimal biological fluid for miRNA measurement, because it is easy to obtain from children compared to other biological fluids. Future research should be focused on exploring more specific clinical outcomes.

## Supplementary Information

Below is the link to the electronic supplementary material.

Figure S1. Quality assessment of individual studies using QUADAS tool (DOCX 14 KB)

## Data Availability

The datasets generated for this study are available on request to the corresponding author.
