# Supplementary material for: miRNAs as biomarkers of autism spectrum disorder: a systematic review and meta-analysis
Source: Eur Child Adolesc Psychiatry. 2023 Feb 3;33(9):2957–90. doi: 10.1007/s00787-023-02138-3 (PMC11424746; doi:10.1007/s00787-023-02138-3)
Supplement: Supplementary file 1 — Figure S1. Quality assessment of individual studies using QUADAS tool (DOCX 14 KB) [file 787_2023_2138_MOESM1_ESM.docx]

Table S1. Quality assesment

| **Estudio** | **Risk of bias** | | | | **Applicability concerns** | | |
| --- | --- | --- | --- | --- | --- | --- | --- |
|  | **Patient selection** | **Index test** | **Reference**  **standar** | **Flow and timing** | **Patient selection** | **Index test** | **Reference**  **standar** |
| Sarachana et al., 2010 |  |  |  |  |  |  |  |
| Seno et al., 2011 |  |  |  |  |  |  |  |
| Frye et al. 2021 |  |  |  |  |  |  |  |
| Mor et al., 2015 |  |  |  |  |  |  |  |
| Ander et al., 2015 |  |  |  |  |  |  |  |
| Wu et al., 2016 |  |  |  |  |  |  |  |
| Nguyen et al., 2018 |  |  |  |  |  |  |  |
| Almehmadi et al, 2019 |  |  |  |  |  |  |  |
| Nguyen et al., 2016 |  |  |  |  |  |  |  |
| Vasu et al., 2014 |  |  |  |  |  |  |  |
| Huang et al., 2015 |  |  |  |  |  |  |  |
| Kichukova et al., 2017 |  |  |  |  |  |  |  |
| Cirnigliaro et al., 2017 |  |  |  |  |  |  |  |
| Jyonouchi et al., 2017 |  |  |  |  |  |  |  |
| Vacaro et al., 2018 |  |  |  |  |  |  |  |
| Yu et al., 2018 |  |  |  |  |  |  |  |
| NT et al., 2018 |  |  |  |  |  |  |  |
| Nakata et al., 2019 |  |  |  |  |  |  |  |
| Jyonouchi et al., 2019 |  |  |  |  |  |  |  |
| Ozkul et al., 2020 |  |  |  |  |  |  |  |
| Atwan et al., 2020 |  |  |  |  |  |  |  |
| Zamil et al., 2020 |  |  |  |  |  |  |  |
| Hicks et al., 2016 |  |  |  |  |  |  |  |
| Hicks et al., 2018 |  |  |  |  |  |  |  |
| Hicks et al., 2019 |  |  |  |  |  |  |  |
| Sehovic et al., 2020 |  |  |  |  |  |  |  |
| Ragusa et al., 2020 |  |  |  |  |  |  |  |
|  | | | **High Risk** | **Low Risk** | **Uncertain Risk** |  | |

**Annex 1. Development of the QUADAS-2 for the 19 studies analyzed in the systematic review.**

**Phase 1:** Review question

Ideally children patients can also be adults with autism spectrum disorder

(scope, intended use of the test, index, presentation, pretests):

Index test (s): Detection of microRNA from body fluids (saliva, serum, lymphoblastoid cells, cerebral cortex, cells of the olfactory mucosa) by microarrays, qRT-PCR and sequencing.

Benchmark tests

- Medical history review
- DSM-5 Diagnostic and Statistical Manual of Mental Disorders, Fifth Edition
- Vineland Adaptive Behavior Scale
- EDUS Developmental Behavior Scales (EDUS-DBS)
- ADOS Autism Diagnostic Observation Program
- ADI-R Autism Diagnosis Interview
- International Classification of Diseases ICD-10
- CARS Childhood Autism Rating Scale
- Gilliam GARS Autism Rating Scale
- Wechsler WAIS Intelligence Scale
- SRS social response scale
- Modified Autism Early Detection Questionnaire (for children 1 to 3 years old) with MCHAT-R Follow-up Interview

Target condition: Patients who do not have developmental disorders including ASD

**DOMAIN 1: PATIENT SELECTION**

**A. Risk of Bias**

Describe methods of patient selection:

- Was a consecutive or random sample of patients enrolled? Yes/No/Unclear
- Was a case-control design avoided? Yes/No/Unclear
- Did the study avoid inappropriate exclusions? Yes/No/Unclear

Could the selection of patients have introduced bias? RISK: LOW/HIGH/UNCLEAR

**B. Concerns regarding applicability**

**D**escribe included patients (prior testing, presentation, intended use of index test and setting):

- Is there concern that the included patients do not match the review question?

CONCERN: LOW/HIGH/UNCLEAR

**DOMAIN 2: INDEX TEST(S)**

If more than one index test was used, please complete for each test.

**A. Risk of Bias**

Describe the index test and how it was conducted and interpreted:

- Were the index test results interpreted without knowledge of the results of the reference standard?Yes/No/Unclear
- If a threshold was used, was it pre-specified? Yes/No/Unclear

Could the conduct or interpretation of the index test have introduced bias? RISK: LOW /HIGH/UNCLEAR

**B. Concerns regarding applicability**

- Is there concern that the index test, its conduct, or interpretation differ from the review question?

CONCERN: LOW /HIGH/UNCLEAR

**DOMAIN 3: REFERENCE STANDARD**

**A. Risk of Bias**

Describe the reference standard and how it was conducted and interpreted:

- Is the reference standard likely to correctly classify the target condition?Yes/No/Unclear
- Were the reference standard results interpreted without knowledge of the results of the index test? Yes/No/Unclear

Could the reference standard, its conduct, or its interpretation have introduced bias? RISK: LOW /HIGH/UNCLEAR

**B. Concerns regarding applicability**

- Is there concern that the target condition as defined by the reference standard does not match the review question?

CONCERN: LOW /HIGH/UNCLEAR

**DOMAIN 4: FLOW AND TIMING**

**A. Risk of Bias**

Describe any patients who did not receive the index test(s) and/or reference standard or who

were excluded from the 2x2 table (refer to flow diagram):

Describe the time interval and any interventions between index test(s) and reference standard:

- Was there an appropriate interval between index test(s) and reference standard? Yes/No/Unclear
- Did patients receive the same reference standard? Yes/No/Unclear
- Were all patients included in the analysis? Yes/No/Unclear

Could the patient flow have introduced bias? RISK: LOW /HIGH/UNCLEAR
